# Surgical Interventions for the Management of Obesity-Related Joint Pain: A Narrative Review

**DOI:** 10.7759/cureus.59082

**Published:** 2024-04-26

**Authors:** Mohummed S Alrayes, Mohammed A Altawili, Saud M Alsuabie, Ahmad W Sindi, Kawkab M Alharbi, Kareem M Alsalhi, Randa M Al Alawi, Israa D Ali, Alrashed N Nasser, Jehad M Alabdulrahim, Mohammed H Alkhaldi, Hamad M Alhudhaif, Sultan A Alotaibi

**Affiliations:** 1 General Surgery, King Fahad Specialist Hospital, Tabuk, SAU; 2 General Practice, Al Aziziyah Primary Health Care Center, Tabuk, SAU; 3 Surgery, King Faisal University, Al-Ahsa, SAU; 4 General Practice, King Abdulaziz University Faculty of Medicine, Jeddah, SAU; 5 Surgery, Princess Nourah Bint Abdulrahman University, Riyadh, SAU; 6 Surgery, Batterjee Medical College, Jeddah, SAU; 7 Surgery, Umm Al-Qura University, Al Qunfudhah, SAU; 8 Surgery, Saudi German Hospital, Riyadh, SAU; 9 General Practice, Imam Abdulrahman Bin Faisal University, Dammam, SAU; 10 Surgery, Qassim University, Al Qassim, SAU; 11 General Practice, King Saud Medical City, Riyadh, SAU; 12 General Practice, Imam Mohammad Ibn Saud Islamic University, Riyadh, SAU; 13 Surgery, King Abdulaziz University, Jeddah, SAU

**Keywords:** surgical management, arthroscopy, osteoarthritis, gastrectomy, pain, obesity

## Abstract

Obesity-related joint pain is a common and debilitating condition that significantly impacts the quality of life, primarily due to the excess weight straining the joints. This results in inflammation and degeneration, which can cause pain, stiffness, and difficulty moving. We aimed to comprehensively review the literature discussing surgical interventions for obesity-related joint pain. We searched across databases (PubMed, Scopus, and Cochrane Library) to identify studies published between 2000 and 2023 that assessed surgical interventions for obesity-related joint pain. This review highlights the complex interplay of mechanical, inflammatory, and metabolic factors contributing to joint pain in obese individuals, highlighting both surgical and non-surgical interventions. Non-surgical interventions include weight loss, exercise, physical therapy, and medications. Surgical interventions include bariatric surgery and joint replacement surgery. Bariatric surgery significantly reduces body weight and improves the quality of life outcomes; however, multiple studies have found no improvement or worsening of joint pain post-surgery. Total joint arthroplasty has demonstrated good improvement in pain and function outcomes based on recent meta-analyses, although risks of complications are higher in obese patients. The treatment choice for obesity-related joint pain depends on the individual patient’s circumstances. Non-surgical interventions are usually the first line of treatment. However, if these interventions are not effective, surgical interventions may be an option.

## Introduction and background

Obesity is a chronic, abnormal, excessive body fat accumulation affecting the health and quality of life. Individuals with a body mass index (BMI) over 30 kg/m^2^ are considered obese [1]. Globally, about one billion individuals are obese, with more than four million people dying each year due to being overweight or obese [1,2]. Obesity may affect any age and body system causing a wide range of non-communicable diseases. Obesity increases the risk of cardiovascular disease, type Ⅱ diabetes, mental health issues, and various forms of cancer [2]. The musculoskeletal system has to bear this excessive body weight throughout the lifespan of obese individuals. The majority of obese individuals suffer from joint pain. Joint pain severity increases with the increase in BMI [3]. The load-bearing joints are the most commonly affected joints such as the lower back and lower limb joints [3,4]. Moreover, upper limb joints may manifest pain in obese individuals [5].

Joint pain could represent the underlying pathological process of osteoarthritis which is the main adverse effect of obesity on the musculoskeletal system [6]. Osteoarthritis is a joint degenerative clinical syndrome represented by joint pain and dysfunction. Gaining 5 kg of weight is correlated with a 36% higher risk of developing osteoarthritis [7]. Chen et al. reported that 50% of patients who required total knee replacement due to end-stage osteoarthritis were obese [8].

There are several management options for addressing obesity. The right option is determined according to the patient’s needs and health condition. Weight reduction methods vary between dietary modification, physical exercise, pharmacological methods, and bariatric surgery. Weight loss of 5-10% of total body weight has been found to improve pain, self-reported disability, and physical quality of life in obese patients with knee osteoarthritis (mild-to-moderate obesity) [9].

Meaningful weight loss, typically defined as a reduction of at least 5% of initial body weight over a six-month period, is a challenging lifestyle modification versus bariatric surgery. Bariatric surgeries could provide 20% weight loss of a patient’s body weight if the patient follows the postoperative instructions. Bariatric surgeries include several techniques such as gastric bypass, sleeve gastrectomy (SG), adjustable gastric banding, and vertical banded gastroplasty. BMI reduction range of 6.2-14.7 kg/m^2^ was found to be associated with knee and back pain relief in 5-100% of obese patients and reduced pain severity in 31-94% of patients with other joints [10]. This review aims to summarize the different surgical management options for obesity-related joint pain and postoperative outcomes.

## Review

Methodology

We searched PubMed, Scopus, and Cochrane Library to identify papers published between 2000 and 2023. Search terms included “obesity,” “joint pain,” “osteoarthritis,” “surgical interventions,” “bariatric surgery,” “sleeve gastrectomy,” “adjustable gastric banding,” “gastric bypass,” “joint replacement,” and “arthroplasty.”

Mechanisms of obesity-related joint pain

Mechanical Overload

Obesity, defined as a BMI over 30 kg/m^2^, presents a significant risk for the development of knee osteoarthritis, which primarily manifests as joint pain [1,11]. The link between obesity and increased joint pain is also reflected in the association between higher BMI levels, elevated pain scores, reduced mobility, and lower physical activity [12].

The knee, as a weight-bearing joint, is particularly susceptible to the mechanical stressors associated with excess body weight [8]. Increased mechanical load can disrupt the delicate balance of cartilage homeostasis, leading to a cascade of degenerative changes within the joint structure [8]. Animal studies have illustrated that excessive weight can cause the cartilage to thin, degenerate more rapidly, and affect the underlying subchondral bone, potentially leading to increased bone thickness and the formation of bone marrow lesions [13,14].

Another aspect of mechanical stress involves changes in body composition, such as increased thigh girth in obese individuals. This can lead to altered biomechanics, including greater hip abduction and a knee varus deformity [8,12]. Such changes result in an uneven distribution of load across the knee joint, particularly along the medial compartment, where the articular cartilage is more prone to early damage due to these altered loading patterns [8].

Although the direct mechanical impact of excess body weight on weight-bearing joints is clear, there is also evidence of obesity contributing to degeneration in non-weight-bearing joints such as the hands [12]. This indicates that mechanical factors alone do not fully explain the association between obesity and knee joint pain, suggesting that systemic effects, possibly inflammatory or metabolic, also play a significant role [12].

The excess body weight associated with obesity increases the mechanical stress on weight-bearing joints, which contributes to the deterioration of cartilage and the development of osteoarthritis. Studies have shown that morbid obesity causes early lesions of the knee cartilage, increased bone matrix micro-damage and altered vasculature in the subchondral bone of bone marrow lesions, increased incidence of knee osteophytes, higher incidence of meniscal extrusion, marked fibrosis with increased macrophage infiltration of the synovium, and Increased incidence of horizontal fissuring [15-20].

Inflammation

**Figure 1:**
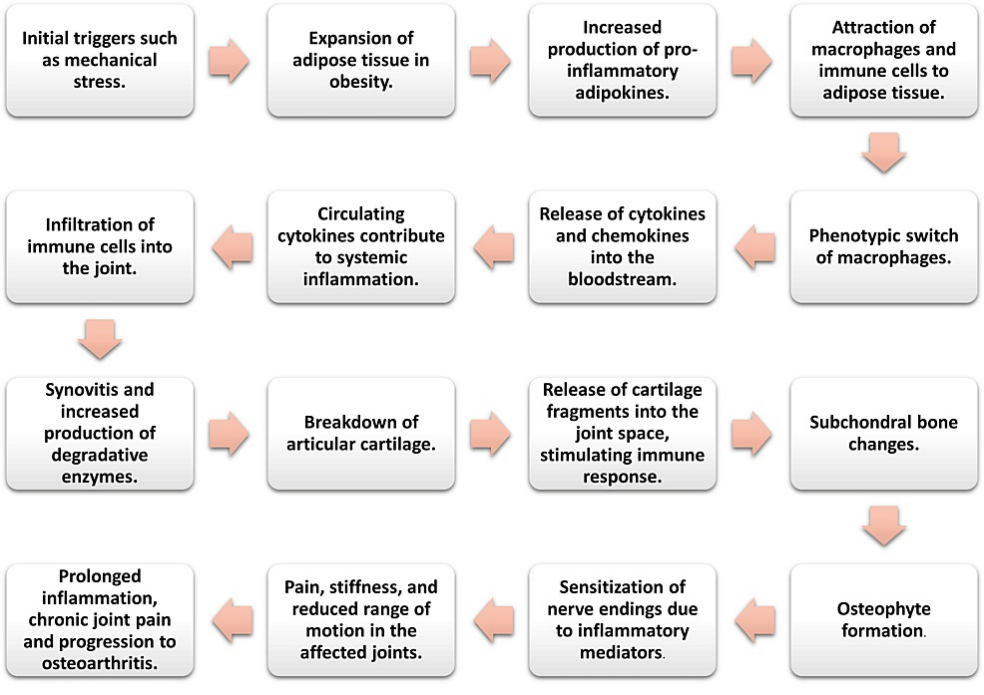
Flowchart of inflammatory pathways in obesity-related joint pain. Authors’ own image.

Contemporary investigations have delineated a significant inflammatory component in obesity-associated joint pain, particularly attributing adipose tissue as a prolific source of various inflammatory mediators, including cytokines, chemokines, and adipokines [21] (Figure 1). These adipocyte-derived factors can exert dual actions, either promoting cartilage catabolism or aiding in its structural maintenance, via intricate molecular pathways [21,22].

Accumulation of adipose tissue, especially visceral fat, which is the fat surrounding internal organs, is not just harmful in the way that it increases the risk of heart disease and conditions such as diabetes, but it also secretes multiple pro-inflammatory cytokines, including interleukin-6 (IL-6), tumor necrosis factor-alpha (TNF-α), and adipokines such as leptin [23-26]. The accumulation of these inflammatory substances leads to a chronic low-grade inflammatory state known as adipose tissue inflammation, which can lead to systemic inflammation and contribute to joint pain [27] (Table 1).

**Table 1:** Adipokines and cytokines: mediators of inflammation and degradation in osteoarthritis and obesity [20-26,28-40]. BMI = body mass index; IL-6 = interleukin-6; MMPs = matrix metalloproteinases; OA = osteoarthritis; TNF-α = tumor necrosis factor-alpha

Adipokine/Cytokine	Effect on joint	Associated changes in obesity
IL-6	Pro-inflammatory, contributing to systemic and joint inflammation	Higher levels in obesity, exacerbating OA
TNF-α	Pro-inflammatory, involved in joint degradation	Increased secretion by M1 macrophages in obesity.
Leptin	Promotes pain and cartilage breakdown and induces pro-inflammatory responses	Serum levels rise with BMI and decrease with weight loss
Adiponectin	Dual role: can promote osteophytes but also has anti-inflammatory effects	Elevated in knee OA and can modulate joint homeostasis
Resistin	Stimulates inflammatory mediators and enzymes involved in cartilage catabolism	Higher serum levels linked to OA severity
Visfatin	Upregulates inflammatory mediators and enzymes involved in cartilage catabolism	Levels correlate with joint damage and OA symptoms

Remarkably, obesity instigates an immunological shift within the macrophage populations residing in adipose tissues. Normally anti-inflammatory and reparative M2 macrophages transition towards a pro-inflammatory M1 phenotype under obese conditions, which, in turn, augments the secretion of inflammatory cytokines such as TNF-α, IL-6, and IL-1β [28]. This phenotypic switch contributes to an inflammatory milieu conducive to joint degradation.

Adiponectin, an adipokine paradoxically elevated in the serum of individuals with knee osteoarthritis, has been implicated in both the development of osteophytes and joint space narrowing, aligning with an increased radiographic grading of osteoarthritis severity [29]. Yet, adiponectin also possesses anti-inflammatory attributes, evidenced by its ability to shift macrophages from an M1 to an M2 phenotype and by its reduction of TNF-α levels [30]. Moreover, adiponectin is implicated in the reduction of systemic lipid-induced oxidative stress and modulation of joint homeostasis by upregulating tissue inhibitor of metalloproteinases 2 and inhibiting the IL-1β-induced expression of matrix metalloproteinase 13 (MMP-13), both of which are critical in maintaining extracellular matrix integrity [31,32].

Leptin, another prominent adipokine, has been observed at elevated serum levels in individuals with higher BMI, correlating with increased pain (as measured by the Western Ontario and McMaster Universities Osteoarthritis Index (WOMAC) index) and joint structural damage [33]. Conversely, leptin levels decrease following weight loss, aligning with symptomatic improvement [34]. In osteoarthritic joints, chondrocytes exhibit heightened leptin production, which, in turn, escalates the release of a suite of pro-inflammatory cytokines and MMPs, further contributing to cartilage breakdown [35,36]. Furthermore, leptin acts on synovial fibroblasts and osteoblasts to induce inflammatory responses and subchondral bone changes [37].

Resistin, another adipokine, has been implicated in the exacerbation of osteoarthritis severity, stimulating chondrocytes to produce MMPs and pro-inflammatory cytokines, thereby fostering joint structural impairment [38,39]. Elevated serum levels of resistin in osteoarthritis patients are associated with the presence of cartilage defects, bone marrow lesions, and heightened pain, along with more pronounced structural joint changes [39].

Visfatin, a pro-inflammatory adipokine, plays a role in osteoarthritis pathogenesis by upregulating inflammatory mediators such as TNF-α, IL-6, IL-1β, and various MMPs and ADAMTS enzymes, which are principal agents in cartilage catabolism. Visfatin’s levels in serum and synovial fluid are positively correlated with joint damage, biochemical markers of collagen type II and aggrecan degradation, C-reactive protein levels, and the symptomatic manifestations of osteoarthritis [40].

Metabolic Factors

Obesity’s influence on joint pain extends beyond mere mechanical stress; metabolic factors play a critical role in the development of osteoarthritis. Obesity is often related to a group of metabolic disturbances known as metabolic syndrome, which includes insulin resistance, type 2 diabetes mellitus, hypertension, and dyslipidemia [41,42]. These metabolic disturbances are associated with an altered composition of synovial fluid and reduced cartilage repair capacity, which contributes to joint pain [43-45].

Dyslipidemia, a common metabolic disturbance in obesity, alongside the resultant lipotoxicity, has recently been recognized as a contributor to osteoarthritis [46,47]. Below we discuss how these metabolic factors intertwine with joint health.

Abnormal lipid profiles can wreak havoc on joints. In osteoarthritis, there is an upswing in the activities of enzymes such as 25-hydroxycholesterol, 7α-hydroxylase, and cholesterol 25-hydroxylase, which appear to be pivotal in joint deterioration. They promote the activity of certain enzymes (MMPs and ADAMTS) known for their role in cartilage breakdown, leading to symptoms such as synovitis, bone spurs, and hardening of the subchondral bone [48].

The destructive duo of reactive oxygen species and free fatty acids can Induce mitochondrial dysfunction in cartilage cells, further accelerating the release of inflammatory cytokines [49-51]. This oxidative stress, compounded by increased levels of cholesterol, triglycerides, and particularly low-density lipoprotein (LDL), has been correlated with more symptomatic knee osteoarthritis [52]. Notably, a higher LDL count has been linked to synovial inflammation and aberrant bone formation, both associated with amplified knee pain [46].

Hypertriglyceridemia and a decrease in protective high-density lipoprotein have been connected to more intense knee pain [53]. The lipid profile of an individual with obesity and osteoarthritis often includes high levels of total cholesterol and LDL, which exacerbate joint inflammation and pain [52].

Sarcopenic obesity, characterized by the coexistence of excess body fat and reduced muscle mass, has emerged as a significant factor associated with knee osteoarthritis [54]. The genesis of sarcopenic obesity is multifaceted, encompassing age-related declines in physical activity, dietary changes, and hormonal alterations. Furthermore, systemic chronic inflammation orchestrated by adipokines accelerates muscle loss, and a higher concentration of leptin, commonly found in sarcopenic obesity, is linked to reduced muscle mass [55].

Adipokines such as leptin and adiponectin are central to the interplay between obesity, muscle mass, and joint pain. Elevated leptin can lead to insulin resistance and systemic inflammation due to leptin resistance and a decline in leptin receptor numbers [55]. Conversely, adiponectin is known to help protect muscle proteins and is typically found in lower levels in obese and insulin-resistant individuals [56]. Consequently, individuals who become sarcopenic may lack sufficient adiponectin to protect against muscle degradation.

Sarcopenia has been discovered in nearly half of all knee osteoarthritis cases, a rate that is double that of healthy controls [57]. The potential mechanism connecting sarcopenia and osteoarthritis may involve impaired sensory input from the affected knee, leading to negative impacts on the motor neurons that control quadriceps muscles. This results in quadriceps weakness, which is a known risk factor for the progression of knee osteoarthritis and related pain [57].

Pain generation

Obesity-related joint pain, particularly in osteoarthritis, is a complex interplay between mechanical load and biochemical processes. While articular cartilage cannot generate pain due to the lack of blood vessels and nerve endings, other structures within and around the joint are rich in sensory nerves and can be sources of intense pain.

Innervation of Joint Structures and Nociceptive Pain

The synovium, joint capsule, ligaments, subchondral bone, and Hoffa’s fat pad are well-innervated and can produce nociceptive pain, a type of pain that arises from actual or threatened damage to non-neural tissue and is due to the activation of nociceptors. These nociceptors respond to mechanical, chemical, or thermal stimuli and release neuropeptides such as substance P and calcitonin gene-related peptides, which are key players in pain signaling [58].

When these nerve terminals are activated, they release a cascade of signals that are transmitted to the dorsal root ganglia and then to the spinal cord. This sets off an inflammatory response involving the NLRP3 inflammasome, CCL2/CCR2 signaling, and the Wnt/β-catenin pathway [59]. The pain signal then travels through ascending pathways to the central nervous system where it is registered as conscious pain [60-62].

Neuropathic Pathways and Obesity-Related Joint Pain

Apart from the direct damage to tissues, neuropathic pain, which arises from lesions or diseases affecting the somatosensory nervous system, can play a role in osteoarthritis pain. Cytokines such as TNF-α, IL-6, IL-1β, and nerve growth factor, as well as chemokines such as CCL2, contribute to peripheral sensitization of sensory nerve fibers [63]. Persistent pain stimuli can result in central sensitization, leading to hyperalgesia (increased sensitivity to pain) and allodynia (pain due to a stimulus that does not normally provoke pain), which are hallmarks of neuropathic pain.

Non-surgical management of obesity-related joint pain

Weight Loss and Exercise

**Figure 2:**
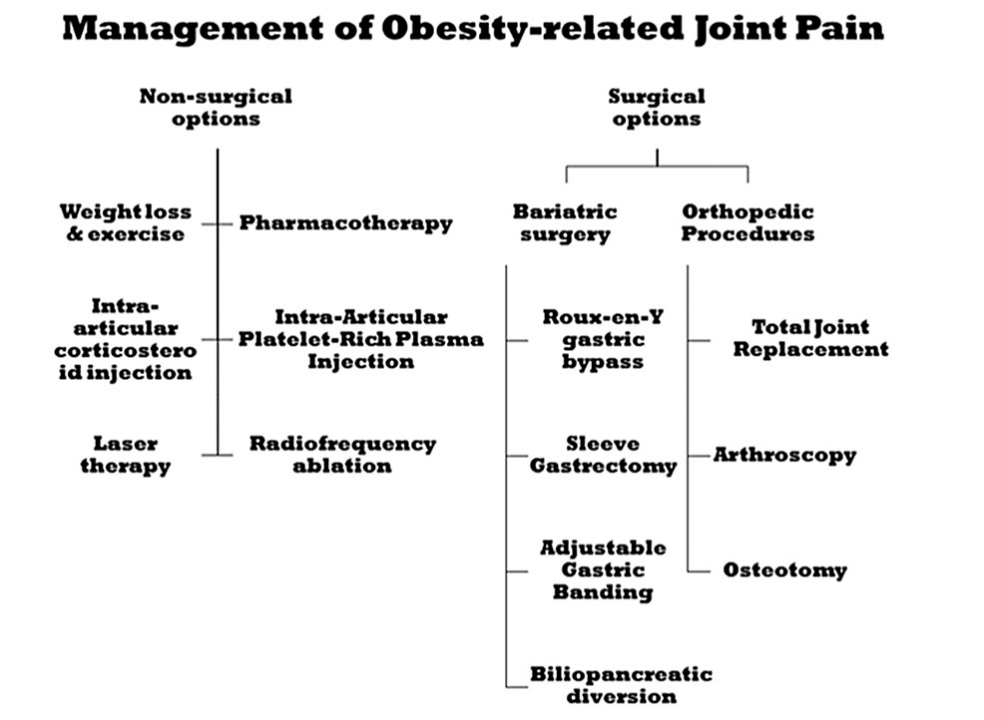
Surgical and non-surgical interventions for the management of obesity-related joint pain. Authors’ own image.

Weight is a cornerstone in the non-surgical approach to joint pain in obese patients (Figure 2). Losing ≥5% of body weight can significantly improve clinical outcomes, and the benefits continue to increase with more weight reduction [64]. Weight loss can be promoted by a low-impact exercising program, which can also help improve joint mobility and strengthen muscles around joints [64,65].

Weight loss plays a pivotal role in managing joint pain for individuals suffering from obesity-related osteoarthritis. The excessive mechanical load on weight-bearing joints due to excess body weight is a primary factor in the acceleration of cartilage degradation. By reducing body weight, patients can significantly decrease the stress on their joints, potentially slowing the progression of osteoarthritis and reducing pain [8]. Beyond the mechanical benefits, weight loss can mitigate the chronic low-grade inflammation associated with obesity, as adipose tissue is known to secrete pro-inflammatory cytokines that contribute to joint inflammation. Significant weight loss, particularly between 10% and 20% of baseline body weight, has been shown to enhance clinical outcomes and reduce pain more effectively than more modest weight loss [66].

Dietary interventions play a critical role in this process. A study of 89 obese patients with knee osteoarthritis who followed a low-calorie diet and received regular dietary counseling exhibited a mean weight loss of 10.9 kg and a statistically significant decrease in their WOMAC pain scores, demonstrating the direct impact of weight management on joint pain [67]. Structurally, weight loss has been associated with increased proteoglycan content in the joint’s extracellular matrix and a reduction in the rate of cartilage thickness loss, as well as favorable changes in biochemical markers indicative of cartilage synthesis and degradation [68,69].

Incorporating exercise, especially programs aimed at strengthening the quadriceps, has also been shown to decrease knee pain. A randomized control trial (RCT) involving 289 obese individuals demonstrated that a two-year exercise program focused on quadriceps strengthening was associated with a statistically significant reduction in knee pain [70]. Resistance training is particularly important, as it helps to improve muscle strength, which, in turn, enhances joint stability and mobility, providing pain relief in a significant proportion of patients with obesity-related knee osteoarthritis [8].

Furthermore, weight loss and exercise are essential for managing traditional cardiovascular risk factors, which is crucial given the established connection between cardiovascular health and musculoskeletal function [6]. Therefore, a comprehensive approach to weight loss that combines dietary changes with an appropriate exercise regimen is not only beneficial for joint pain management but also for overall health and well-being.

Pharmacotherapy

Non-steroidal anti-inflammatory drugs (NSAIDs) are recommended drugs that can help reduce pain and inflammation. NSAIDs function by inhibiting the COX enzyme, which is responsible for the conversion of arachidonic acid to prostaglandins and thromboxane, thereby reducing the inflammatory response [71]. A meta-analysis of 76 randomized trials identified diclofenac 150 mg/day as the most effective NSAID in the management of joint pain [72]. The long-term use of NSAIDs carries an increased risk of side effects such as gastrointestinal bleeding, up to four times, as well as an increased risk of cardiovascular complications [73,74]. However, compared to non-selective oral NSAIDs, certain formulations such as meloxicam and naproxen combined with lansoprazole have been shown to have a lower risk of adverse gastrointestinal events, indicating that some NSAIDs may be safer for long-term use [73,74]. Topical NSAIDs, such as diclofenac gels and patches, have been shown to produce more efficacy with lesser toxicity, and are widely recommended as an early treatment option as they provide similar efficacy to oral NSAIDs with fewer side effects [75]. They can lower the need for oral NSAIDs by 40% [75]. Furthermore, studies indicate that NSAIDs may increase matrix synthesis and protect chondrocytes against apoptosis, potentially aiding in the regeneration of articular cartilage function [76]. Because of their efficacy in managing both acute and long-term musculoskeletal pain, NSAIDs, and specifically diclofenac gel, have emerged as commonly used medications for the treatment of musculoskeletal disorders, which have a significant impact on individual lifestyles and society [77].

Intra-articular Corticosteroid Injection

Corticosteroids are well-known as potent anti-inflammatory agents. They act on nuclear receptors, disrupt the inflammatory cascade, and reduce the action and production of ILs, prostaglandins, and leukotrienes [78]. Evidence proves that corticosteroid injections have significantly higher efficacy compared to that of other drugs [64].

Intra-articular Platelet-Rich Plasma Injection

Platelet-rich plasma (PRP) is a safe and efficient therapy for osteoarthritis. It triggers cartilage regeneration and promotes growth factors such as platelet-derived growth factor, insulin-like growth factor, vascular endothelial growth factor, and fibroblast growth factor. These factors play a role in the proliferation and differentiation of cells. PRP can also reduce adipogenesis and inflammation in infrapatellar fat pad [79,80]. This meta-analysis indicated that PRP injections were effective in reducing pain symptoms. In addition, PRP injection therapy can effectively improve the functional activity of osteoarthritis patients and has a high level of safety for clinical applications [81].

Laser Therapy

Laser therapy, also known as low-level laser therapy or cold laser therapy, is a therapeutic modality that involves the application of low-intensity lasers or light-emitting diodes to target tissues, stimulating cellular activity and promoting healing processes. Studies have shown that a combination of laser therapy with exercise was significantly more effective in reducing pain in comparison to exercise alone or placebo [82]. A systematic review of 10 studies found both low-level laser therapy and high-intensity laser therapy were effective in reducing knee joint pain symptoms. Both showed significant improvements in knee pain, stiffness, and function for high-intensity laser therapy [82].

Radiofrequency Ablation

Radiofrequency ablation (RFA) is a procedure that involves the use of radiofrequency (RF) energy to create localized heat, which targets specific nerves responsible for transmitting pain signals from affected joints to the brain. By targeting these nerves, RFA disrupts their ability to transmit pain, offering pain relief. In the study conducted by Liu et al., a comprehensive meta-analysis was performed to assess the effectiveness and safety of RF treatment for knee osteoarthritis. The analysis included 15 RCTs with a total of 1,009 patients. Liu et al.’s study indicated that RF treatment was associated with significant improvements in pain relief, as evidenced by reduced scores on the Visual Analog Scale/Numeric Rating Scale, and enhanced knee function, as demonstrated by improved WOMAC pain scores. These improvements were observed at multiple follow-up points, specifically at 1-2, 4, 12, and 24 weeks after treatment [83]. Additionally, patients reported a high degree of satisfaction with the effectiveness of RF treatment, measured by the Global Perceived Effect scale at 12 weeks, with the results reaching statistical significance (all p < 0.001). The Oxford Knee Score, which is another measure of knee function, did not show a significant difference between groups treated with RF and those that were not. Importantly, the analysis also found that RF treatment did not lead to a significant increase in adverse effects, suggesting that it is a safe option for patients with knee osteoarthritis. A subgroup analysis specifically examining knee pain revealed that RF treatment targeting the genicular nerve had significantly better efficacy than intra-articular RF at the 12-week post-treatment mark (p = 0.03). The conclusions drawn from this meta-analysis by Liu et al. suggest that RF treatment is both an effective and safe modality for providing relief from knee pain and improving knee function in patients suffering from knee osteoarthritis [83].

The study by Zhang et al. (2021) centered on evaluating the efficacy and safety of RFA in the treatment of knee osteoarthritis [84]. Their meta-analysis incorporated data from nine RCTs. The results of their study highlighted that there were significant differences in pain scores when comparing patients who received RFA to those who were given a placebo [84]. These differences were consistently observed at various post-treatment intervals, specifically at 4, 12, and 24 weeks [84]. In addition to the observed improvements in pain, Zhang et al. also reported that patients undergoing RFA experienced better outcomes in knee function, as measured by the WOMAC pain scores. These improvements were noted at the same follow-up intervals of 4, 12, and 24 weeks, indicating sustained benefits over time [84]. Another essential aspect of their findings was the safety profile of RFA. The study found that no serious adverse events were reported in patients who underwent this treatment, implying that RFA is a relatively low-risk option for individuals with knee osteoarthritis [84]. Overall, Zhang et al. supported the use of RFA as an effective and safe therapeutic approach for reducing pain and improving knee function in patients with knee osteoarthritis, without an increased risk of adverse effects [84].

Surgical interventions for obesity-related joint pain

Bariatric Surgery

A meta-analysis of 20-year outcomes after bariatric surgery, which included 33 studies on patients who underwent Roux-en-Y gastric bypass (RYGB), biliopancreatic diversion with or without duodenal switch (BPD/DS), and laparoscopic adjustable gastric banding found that BPD/DS produced significantly greater weight loss compared to all other operation types, with a mean of 74.1% of excess weight loss, followed by RYGB with 56.7% excess weight loss than adjustable gastric banding with 45.9% excess weight loss [85]. However, there was no difference in weight, loss between RYGB and SG. Weight loss at 20 years was 30.1 kg, 48.9% of excess weight lost, and 22.2% total weight loss. The reoperation rate was initially high but reduced markedly with improving band, surgical, and aftercare techniques [85].

Multiple studies suggested that although bariatric surgery contributed to significant weight loss, it did not improve joint pain and in some cases increased it. Rocha et al. (2023) assessed post-bariatric surgery outcomes using the modified Nordic Musculoskeletal Symptoms Structured Questionnaire and the osteoarthrosis-specific quality of life questionnaire WOMAC to evaluate knee pain and demonstrated improved physical performance and overall quality of life, but maintained or increased pain complaint of patients. The study concluded that the physical improvement was due to the reduction of joint overload, with worsening or maintenance of pain [86]. These claims can be supported by multiple previous studies that assessed the chronic use of opioids for pain management before and after the surgery and found an increase in the prevalence of analgesic use after bariatric surgery [87-89]. This can depict increased pain complaints after the surgery. It is also worth noting that Tan et al. (2022) found a decrease in the rate of total joint arthroplasty from 10.8% at baseline to 3.15% after six years of undergoing surgery [89]. King et al. (2017) followed 1,491 patients who underwent RYGB or SG for seven years after the surgery and found improvement in joint pain and physical function ranging from 41% to 72%, but there was no change in back pain medication use before and after the surgery, with some patients not experiencing any improvement after the surgery [88].

Regarding metabolic changes following bariatric surgery, there is a significant improvement in type 2 diabetes mellitus, hypertension, and hyperlipidemia across six years [89].

One of the most serious complications of any bariatric surgery is the risk of anastomotic leaks, as it increases morbidity to 61% and mortality to 15% [90]. A meta-analysis of 29 studies including 4,888 patients treated with SG found the risk of leak to be 2.4%. The risk of anastomotic leak increased in patients with higher BMI, reaching 2.9% in patients with a BMI of <50 kg/m^2^ [91]. A retrospective multicenter analysis of 4,120 RYGB and 1,457 SG low-risk cases demonstrated a risk of leakage of lower than 0.15% and 1.3% in patients undergoing SG and RYGB, respectively, and the incidence of stenosis of anastomosis was 1.2% in RYGB [92].

The incidence of postoperative bleeding was up to 11% of cases in both RYGB and SG in the study by Doumouras et al. (2016), but was only 2.2% and 1.7% in RYGB and SG, respectively, in the study by Gero et al. (2019) [92,93].

Cholelithiasis is a common complication of bariatric surgery, with an incidence of 32.5% and 25.5% in patients undergoing RYGB and SG, respectively. Fortunately, it can be reduced to 5.7% and 2.4% in RYGB and SG, respectively, if patients are treated with ursodeoxycholic acid [94].

Another feared complication is venous thromboembolism. A multicenter study of 4,293 patients identified thromboembolic events in 1.3% of patients, with pulmonary embolism being the most common cause of mortality [95].

One of the complications specific to adjustable gastric banding is band slippage with a reported incidence ranging between 4% and 13% [96].

Furthermore, multiple studies reported nutritional deficiencies after bariatric surgery, most notably vitamin D, iron, folate, vitamin B12, and zinc. In the study by Elhag and El Ansari (2022), multiple deficiencies were present among 97.6%, 73.2%, 23.6%, 15%, and 12.6% of adolescents, who had vitamin D, albumin, hemoglobin, zinc, and vitamin B12 deficiencies, respectively. Hasan et al. (2020) found iron, mean corpuscular volume, hemoglobin, and ferritin deficiencies (48.7%, 47.0%, 46.7%, and 38%, respectively), and 40% and 41.3% had 25-OH vitamin D insufficiency and deficiency, respectively. Further, De Sousa Paredes and Mota-Garcia (2020) and Ben-Porat et al. (2020) reported similar results [97-100].

Dumping syndrome occurs because the stomach contents are rapidly dumped into the small intestine without proper digestion, leading to a surge in blood sugar levels and subsequent changes in bodily functions. A prevalence study in Saudi Arabia investigated 240 cases who underwent bariatric surgery and found that 31.4% of the patients met the criteria for dumping syndrome. The study identified gender, educational level, and eating habits as predictors of the syndrome [101].

Orthopedic Procedures

There is a scarcity of high-quality evidence regarding joint replacement procedures in obese patients. However, a recent systematic review and meta-analysis included 31 studies with 103,451 cases who underwent a total hip replacement and 153,098 cases who underwent a total knee replacement. For total hip replacement, the Hip Disability and Osteoarthritis Outcome Score (HOOS) improved from a baseline of 41.55 to 87.09, and the HOOS pain score improved from 44.22 to 90.94, and from 46.94 and 26.64 to 88.89 and 80.3 on the HOOS score of activities of daily living (ADL) and quality of life, respectively [102].

For total knee replacement, the Knee Disability and Osteoarthritis Outcome Score (KOOS) increased from a baseline of 45.58 to 80.63 and the KOOS pain score increased from 48.14 to 86.8, while the KOOS ADL and quality of life scores increased from 54.74 and 27.41 to 86.04 and 71.9, respectively [102].

Although this meta-analysis did not classify patients depending on obesity status, the results can be considered as a recent systematic review by Courtine et al. (2023) stated that the outcomes of total hip replacement and total knee replacement did not differ significantly in patients with obesity compared to those without it [103].

Total joint replacement, as with any other surgery involving the implantation of a foreign body, carries a risk of complications. A recent study analyzed data from 5,153 patients with a mean age of 56.7 ± 7.8 years and found that 1% of patients underwent revision and 1.4% were readmitted within 90 days of the surgery. The most common causes for revision were infection in 20.8% of revised cases, followed by instability in 15.1%, periprosthetic fracture in 13.2%, and aseptic loosening in 9.4%. The most common cause of readmission was infection in 22.9% of readmitted cases, followed by pain in 9.5% and per prosthetic fracture in 5.4% [104]. The risk of complications increases with age, as demonstrated by Yohe et al. (2020) in a study of 7,730 patients over the age of 80 [105]. The study found that 25.1% of patients experienced minor complications. Minor complications included superficial wound infection, acute renal failure, deep venous thrombosis, peripheral neurological deficit, and pneumonia. The occurrence of bleeding requiring transfusion occurred in 22.9% of patients, while 4.9 experienced major complications such as deep wound infection, wound dehiscence, cerebral vascular accident, pulmonary embolism, myocardial infarction, cardiac arrest, and sepsis [105].

Compared to bariatric surgeries the risk of gastrointestinal complications due to total joint replacement is low. In a recent study, a cohort of 36,932 patients who underwent total hip arthroplasty or total knee arthroplasty found the risk of gastrointestinal complications to be 0.75%, with the risk of severe ileus or gastrointestinal bleeding to be as low as 0.09% [106].

Arthroscopy

A systematic review and meta-analysis by Kuroda et al. (2021) analyzed the data from eight studies and found that the modified Harris Hip Score was 5.1 points lower in the obese group while the Non-arthritic Hip Score was nine points lower in the obese group [107]. Moreover, the odds ratios for revision arthroscopy, conversion to total hip replacement, and complications were 1.2, 2.4, and 3.2, respectively. Another systematic review and meta-analysis by Zhang et al. (2021) assessed the results of 16 articles on obese patients undergoing arthroscopic partial meniscectomy and found that obese patients scored lower on the postoperative normalized knee-specific patient-reported outcome, although the differences in clinical improvement or the progression of osteoarthritis were not significant between the BMI groups [108].

Osteotomy

Osteotomy is a surgical procedure where surgeons cut and reshape the bones to correct alignment issues and redistribute forces on the joint to help improve joint pain and joint function [109]. As the use of osteotomy for the treatment of obesity-related joint pain is uncommon, there is a lack of high-quality studies regarding its outcomes among obese patients. However, a retrospective study involving 280 patients who underwent periacetabular osteotomy found no significant differences in outcomes between obese and non-obese patients, and the risk of complications was higher, reaching 22% in the obese group compared to only 3% for non-obese patients [109]. The study classified the severity of the complications according to the modified Dindo-Clavien classification, where the actual risk of grade IV complications including life-threatening complications such as pulmonary embolism or persistent pain requiring total hip replacement was 8% in obese patients compared to 5% in non-obese patients [109]. The risk of grade III complications requiring invasive radiological or surgical intervention such as deep infections, wound hematoma, and postoperative ileus requiring abdominal intervention was 9% and 3% in the obese and non-obese patients, respectively [109]. Another retrospective study by Wirth et al. (2019) assessed the influence of obesity on the outcome after reversed L-shaped osteotomy for hallux valgus (HV) [110]. The study analyzed 810 cases and found no association between the radiological relapse of HV and no evidence of association between BMI and complications. Further evidence is needed to conclude the safety and efficacy of osteotomy for obese patients.

Patient selection and pre-bariatric surgery assessment

**Figure 3:**
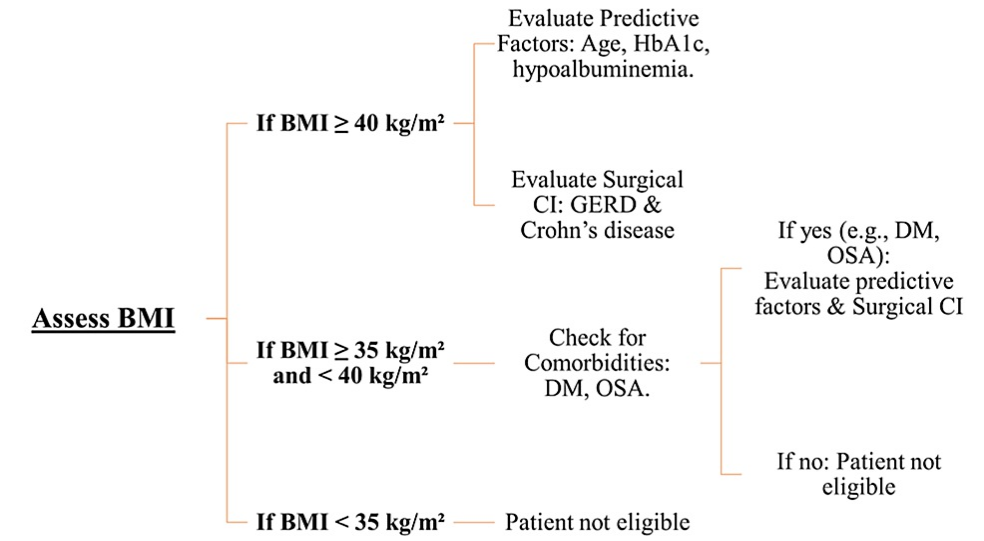
Flowchart for bariatric surgery patient selection according to BMI. Authors’ own image. BMI = body mass index; CI = contraindication; DM = diabetes mellitus; GERD = gastroesophageal reflux disease; OSA = obstructive sleep apnea

Figure 3 presents a flowchart for bariatric surgery patient selection according to BMI. The indication for bariatric surgery is a patient with a BMI higher than 40 kg/m^2^, or a patient with a BMI higher than 35 kg/m^2^ with one or more obesity-related health conditions such as diabetes mellitus and obstructive sleep apnea. For BMIs less than 30 kg/m^2^, bariatric surgery is currently not recommended. A younger age, lower BMI, and lower HbA1c are predictive of higher weight loss after bariatric surgery [111]. Multiple studies have identified hypoalbuminemia as a predictive factor of serious post-surgical complications [112-114]. Lower plasma phospholipid concentration and higher values of total branched-chain amino acids might act as helpful markers for impaired metabolism and metabolic disorders [115].

Small bowel anastomoses, such as RYGB, one anastomosis gastric bypass, or duodenal switch, are contraindicated for patients with Crohn’s disease. Patients complaining of severe gastroesophageal reflux disease symptoms should not undergo SG. RYGB might be a better option as it can help with reflux control [116].

Risk factors for morbidity and mortality should be assessed, which include being male, older than 50 years, having congestive heart failure, peripheral vascular disease, and renal impairment [117,118].

The preoperative assessment should include a psychological assessment as patients undergoing bariatric surgery might have psychological issues that could interfere with the desired outcome of the surgery. A systematic review by Gibbons et al. (2014) found that 25% of patients had depression, 27% had other mood disorders, and 16% had eating disorders. If these issues were not addressed before surgery, they might be a cause for outcome failure and weight regain [119].

Recommendations for future research

Future studies are required to assess the efficacy and safety of surgical interventions for patients with obesity-related comorbidities, such as type 2 diabetes mellitus, hyperlipidemia, and hypertension, who are experiencing obesity-related joint pain. Additional long-term studies are required to evaluate the long-term efficacy of surgical interventions in terms of pain reduction, function improvement, and quality of life. Studies on cost-effectiveness are also required to compare the costs of surgical interventions to other approaches such as weight loss methods and physical therapy.

## Conclusions

This review aimed to explore surgical options for managing obesity-related joint pain, with particular emphasis on bariatric surgeries such as RYGB, SG, and adjustable gastric banding. Additionally, orthopedic procedures, including total joint replacement, arthroscopy, and osteotomy, have been discussed as alternative interventions. By understanding these surgical approaches, healthcare professionals can make informed decisions in the management of obesity-related joint pain, considering the unique needs and circumstances of each patient.

Orthopedic procedures also carry risks of complications. Future research should focus on evaluating the long-term efficacy and safety of surgical interventions, especially in patients with comorbidities. Moreover, future research should compare the cost-effectiveness of surgical interventions with non-surgical approaches such as glucagon-like peptide 1 agonists which promote weight loss.
